# Editorial: Impact of gut ecosystem in health and diseases: microbiome, mucosal barrier and cytokine milieu

**DOI:** 10.3389/fmicb.2023.1345703

**Published:** 2024-01-05

**Authors:** Akihito Harusato, Hirohito Abo, Yoshito Itoh, Timothy L. Denning

**Affiliations:** ^1^Molecular Gastroenterology and Hepatology, Graduate School of Medical Science, Kyoto Prefectural University of Medicine, Kyoto, Japan; ^2^Center for Inflammation, Immunity, and Infection, Institute for Biomedical Sciences, Georgia State University, Atlanta, GA, United states

**Keywords:** microbiome, mucosal barrier, cytokine, health, probiotics, AIEC, colitis associated cancer

Starting at the perinatal period, we encounter a multitude of antigens, foods, pre- and probiotics, and other external factors that shape the development and maturation of the immune system, all while establishing a mutualistic relationship with our microbiota. These environmental components, collectively termed the “exposome,” significantly affect health and disease throughout our lifespan. Especially in the intestine, the interplay between exposome and host immunity regulates epithelial barrier functions as well as cytokine/chemokine production from various immune and non-immune cells. In this rapidly evolving field, new mechanisms by which the exposome maintains intestinal homeostasis are constantly being discovered. In this issue, we highlight the research that provides novel insight into host-microbiota dynamics.

In their review, Gou et al. summarized how probiotics can aid in restoring the intestinal barrier to prevent the entry of harmful substances such as pathogens and endotoxins. They highlight the effects of probiotics on tight junctions as one important mechanism for maintaining homeostasis. The majority of studies have used the cultured cell lines such as Caco-2, IPEC-1, and T84 to demonstrate how probiotics induce the expression of tight junction proteins like ZO-1, claudins, and occludins. Some studies have indicated that probiotics might promote the proliferation of intestinal epithelial cells. Another important mechanism is the modification of mucin barriers by probiotics, including the induction of MUC2 expression by *L. acidophilus A4* and its cell extracts. Additionally, other studies have highlighted probiotic-mediated regulation of immune cells. For example, *Lactobacillus* and *B. infantis* can promote the maturation of dendritic cells. Further, probiotics can compete with pathogens for nutrients, competitively inhibit the attachment sites of targeted cells, or impede the spread of micro-colonies to resist the invasion of pathogens.

Tanaka et al. investigated how the host immune system recognizes and responds to adherent-invasive *E. coli* (AIEC), a pathobiont associated with Crohn's disease. Persistent colonization by AIEC LF82 triggered the secretion of luminal IgA, in contrast to commensal *E. coli* strains. The induced anti-LF82 IgA exhibited specific binding to AIEC strains but not to the commensal *E. coli* strains. Notably, LF82-specific IgA limited the adhesion and invasion of LF82 in cultured epithelial cells. In summary, the authors revealed that host immunity selectively recognizes pathobiont *E. coli*, such as AIEC, and develops specific IgA, thereby preventing these pathobionts from accessing the epithelium.

Wang et al. examined the impact of *A. muciniphila*-mediated post-antibiotic reconstitution of the gut microbiota on a murine model of colitis-associated colorectal cancer (CAC). The results showed that post-antibiotic replenishment of *A. muciniphila* exacerbated CAC tumorigenesis with impaired intestinal barrier function and elevated concentrations of short-chain fatty acids. Despite *A. muciniphila* being recognized for its anti-inflammatory properties, these findings provide an unexpected result. The authors suggest that maintaining the homeostasis of the intestinal microbiota may be more important than replenishing a single microbe.

In the context of the gut ecosystem, it is crucial to understand the interplay between the exposome (such as pathogens, commensals, and dietary additives) and its impact on host immunity and the cytokine milieu. The studies highlighted here provide key new insights into this dynamic. Given the additional complexity of the environmental ecosystem, future studies investigating the effects of components like pollutants/chemicals (e.g., nano and microplastics) on host immunity and the microbiome are likely to provide additional insights.

## Author contributions

AH: Writing—original draft, Writing—review & editing. HA: Writing—review & editing. YI: Writing—review & editing. TD: Writing—review & editing.

